# Prevention of Drowning by Community-Based Intervention: Implications for Low- and Middle- Income Countries

**DOI:** 10.5812/atr.7690

**Published:** 2012-10-14

**Authors:** Ali Davoudi-Kiakalayeh, Reza Mohammadi, Shahrokh Yousefzadeh-Chabok

**Affiliations:** 1 Trauma Research Center, Guilan University of Medical Sciences, Rasht, IR Iran; 2 Department of Public Health Sciences, Division of Social Medicine, Karolinska Institutet, Stockholm, Sweden

**Keywords:** Drowning, Prevention and Control, Intervention, Community Based, Iran

## Abstract

**Background:**

Drowning is a serious but neglected health problem in low-and middle-income countries.

**Objectives:**

To describe the effectiveness of drowning prevention program on the reduction of drowning mortality rates in rural settings at the north of Iran, and guide its replication elsewhere.

**Patients and Methods:**

This interventional design included pre- and post-intervention observations in the rural area of the Caspian Sea coastline without a comparison community. Cross-sectional data were collected at pre- and post-intervention phases. Outcome evaluation was based on a four-year period (March 2005-March 2009) utilizing drowning registry data for the north of Iran.

**Results:**

The implementation program increased the rate of membership in an organization responsible for promoting safety in high risk areas near the Caspian Sea. Compared to a WHO standardized population, drowning incidence in rural areas of the study demonstrated a continuous decrease in age-specific drowning rate among the oldest victims with a gradual decline during the implementation. In the study area, the epidemiological aspects of the study population were exposed and contributing factors were highlighted.

**Conclusions:**

This study showed that the promotion of passive interventions had a greater effect on drowning rate than that of active interventions.

## 1. Background

Drowning is a significant cause of unintentional deaths by injury worldwide, particularly in rural regions of low-and middle- income countries. In fact, several recent research publications showed that drowning along with road traffic accidents and burn injuries were the leading causes of injury and death in these countries, when regional physical as well as socioeconomic environmental factors, and drowning patterns were weighed in (1-5). In Iran, during the year 2005, the majority of drowning deaths among all age groups occurred in unprotected areas of the Caspian Sea (where emergency services were not readily available) and rivers; the victims were males (86.5%), tourists visiting the area (44%), and people under the age of 20 (almost 38%) (6, 7). Interestingly, the estimated number of drowning cases in the study population was much higher than that given by official figures, ranging from 5.26 to 8.25 per 100,000 residents compared to national figure of 4.5 per 100,000 residents (8). In 2005, a drowning prevention program was implemented in the north of Iran as the first community-based intervention project aimed at preventing from drowning in the high risk groups and within more hazardous environments. The program took into account many barriers to establish safe seaside recreational activities encountered by resident and tourist populations in the form of passive and active interventions. The interventions were categorized according to Haddon’s model (9, 10). Intervention with addressing to drowning was not consistently applied in low- and middle-income countries; however, we believe that the present study was the first report about the effectiveness of drowning prevention in contemporary environments of both low- and middle- income countries such as Iran.

## 2. Objectives

The aim of the present study was to report the effectiveness of drowning prevention package with associated interventions by employing estimation from drowning fatality rate before and after the program implementation. The estimated mortality rate in the areas of study was measured using a controlled pre- and post-design during March 2005 - March 2009.

## 3. Patients and Methods

This study was part of a population-based research program of drowning deaths occurring between resident and tourist populations of Guilan and Mazandaran provinces from 20 March 2005 through 20 March 2009 (a period coinciding with the Iranian calendar year). A drowning prevention program was established in March 2005 to improve public awareness of measures, increase supervision, and train community first responders; the program was shown as an effective drowning prevention package with the target of reduction in drowning events at multiple settings. Evaluation of the program was performed in the form of a controlled pre- and post-study protocol which employed pre- and post-interventional observations in a rural area near the Caspian Sea coast line. Cross-sectional pre- and post-intervention data were collected in the study area.

### 3.1. Study Area

Near the Caspian Sea, there are two provinces called as Guilan and Mazandaran. At the beginning of the study, about 4.5 million people were residing in the both provinces, of which about 53% were in urban areas and 47% were residents of rural areas (11). The provincial areas around the Caspian Sea were divided into 40 districts with a common socio-demography. In each district there was one protected swimming area with the access to rescue services. The study area was comprised of rural settings in both provinces of the Caspian Sea coastline, with data collection limited to natural open water regions including rivers, lakes, canals, and wells. “Behvarz” teams (i.e. Iran’s healthcare providers) were recruited in these areas to work one-on-one with staffs of health education programs in training sessions related to identification of drowning hazards. At the time of the study, the age and gender structures in the population of Guilan and Mazandaran provinces were comparable, which did not differ significantly from those of national averages.

### 3.2. Drowning Prevention Package

This intervention package was based on health promotion concepts, and its goals covered of all age groups, a variety of physical and socio-economic environments. The nature of the program implementation was such that it was adapted to the Iranian health care framework and infrastructure and was anchored to the local community networks, representing a cross-sectional group for coordination of the drowning program in situ.

Elements of intervention package in rural settings by the Caspian Sea coastline included:

1) With regard to public health awareness, integration of public health messages into local television and radio was promoted in Guilan and Mazandaran provinces during summer seasons (Passive intervention).

2) Informational programs for healthcare providers (Behvarz groups) in rural setting aimed to train community. First responders were employed to educate clients about drowning risk factors and basic resuscitation techniques with specific emphasis on education of high-risk populations (active intervention).

3) Modification of environmental change through, for example, the elimination of certain water reservoirs (passive intervention).

In the study areas, intervention programs intended to gain recreational behavioral changes; this goal was implemented through public health educational campaigns utilizing posters, pamphlets, and notices at the sites of previously occurred drowning. Their purpose was to inform the local community about circumstances related to drowning incidents in the country to educate people about various means of preventing drowning, and to gather data to facilitate determination of drowning causes.

### 3.3. Definition

Drowning was defined as the process of experiencing respiratory impairment from submersion/immersion in a liquid medium. The details of drowning mortality as encoded in the International Classification of Disease, 10th Revisions (ICD-10) (12) were used. All drowning cases comprised by the scope of our research were included in this study.

### 3.4. Data Sources

Drowning data could be obtained from three sources in Iran including: the Death Registry System (DRS) and Forensic Medicine System (FMS), both of which were based on death certificate data, and a weekly report (WR) obtaining case reports from ambulance excursions, thus allowing for case reports not usually registered in hospitals (6). All three datasets were used in the study. Calculating denominator figure of drowning incidence rates, population counts for year 2005 were considered for “before study” category and the year 2009 for “after study” category. Both population figures were obtained from Iran’s official statistics registry (11) with the exclusion of the tourist population, as there was insufficient denominator data for this group.

### 3.5. Data Analysis

Analyses of outcome measures were based on mortality rates in the interval time between the event and hospital discharge. Comparatively, evaluation of outcome measures was performed three years post-implementation of the intervention project (March 2006-March 2009) in a rural setting of the Caspian Sea coastline. To calculate the age for standardized death rate, we employed a new approach proposed by WHO, a model based on the average age-structure within the populations being compared for the period of 2000 - 2025. Symbolically, the standardized mortality rate for each study population was given by the following equation:

Rs= Σ (rk*wk)

where rk stands for the death rate in age group k in study population, and wk stands for the death rate for age group k in standard population (13).The changes in levels of awareness of rural setting population for prevention of drowning were measured by calculating the mean of score 1 to 10 in a household questionnaire survey.

## 4. Results

### 4.1. Program Implementation

Public health awareness: more than 1200 hours educational program were organized by local TV and about 40,000 pamphlets were distributed in the intervention areas to achieve more than 5 million people. A high rate for awareness of local safety programs was found in the 2008 household survey at the end-line period of program implementation in rural setting compared to the baseline time (12% to 55%).

### 4.2. Program Outcome

**Figure 1:**
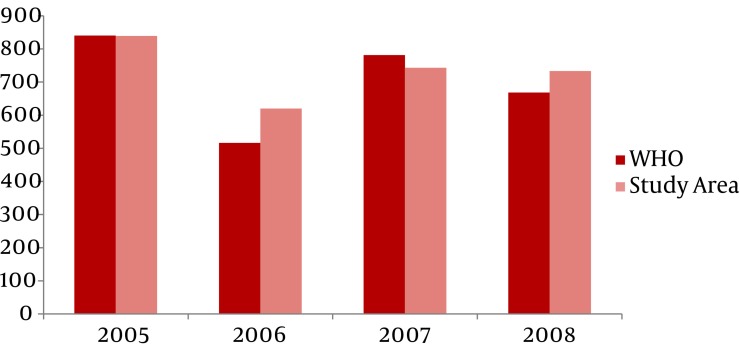
Trend in Age-adjusted Drowning Mortality Rates Based on the Rural Setting of Guilan and Mazandaran Provinces and WHO World Standardized Populations (2005 - 2008)

Overall, the all risk factors associated with drowning incidents declined to a greater extent in the intervention period than the baseline time. In general, most drowning deaths affected men, yet the gender difference in the north of Iran varied slightly across the four-year interval of this study. The ages of 20 and 29 years, respectively, were most likely to drown in the study area. Most drowning fatalities between March 2005 and March 2009 occurred during the summer season (Figure 1).

#### 4.2.1. Rural Setting of the Caspian Sea Coastline

A total number of 381 drowning fatality cases were reported between March 2005 and March 2009. The incident rate fell from 4.5 per 100,000 at baseline time to 3.6 per 100,000 at end-line period, although no consistent trend was detectable. The number of drowning events considered for each individual year of the implementation indicated that the first year (2006) had the lowest rate. The annual trend during the intervention period included an increased drowning fatalities during the second year (2007), a relatively high rate during the year 2007, and another lower rate in the third year of implementation (dome- shaped). During the implementation, the age associated with drowning peaked in the range of 10 to 19 years old with the exception of a temporary elevation in drowning events in the range of 20 - 29 years old during 2007. Sub-grouping by age, according to a WHO world standardized population provided evidence of continued decline in the age-specific drowning rate among victims in the oldest age group (Table 1).

**Table 1:** Standardized Age Group Death Rates from Drowning in Rural Setting of Guilan and Mazandaran Provinces Using WHO World Standardized Populations

	WHO Standardized Population 2005, No. (%)	Study Areas Population 2005, No. (%)	WHO Standardized Population 2006, No. (%)	Study Areas Population 2006, No. (%)	WHO Standardized Population 2007, No. (%)	Study Areas Population 2007, No. (%)	WHO Standardized Population 2008, No. (%)	Study Areas Population 2008, No. (%)
**Age, y**								
0 - 4	115 ( 8.9)	76 (5.8)	53 (8.9)	35 (5.8)	168 (8.8)	114 (6)	44 (8.8)	20 (6)
5 - 9	96 (8.7)	70 (6.4)	43 (8.7)	31 (6.2)	61 (8.7)	44 (6.2)	104 (8.7)	74 (6)
10 - 14	95 (8.6)	95 (8.6)	43 (8.6)	40 (8)	69 (8.6)	61 (7.6)	26 (8.6)	21 (7)
15 - 19	135 (8.47)	192 (12)	101 (8.5)	138 (11)	59 (8.5)	78 (11)	110 (8.5)	136 (10)
20 - 24	66 (8.2)	96 (12)	82 (8.2)	111 (12)	156 (8.2)	229 (12)	106 (8.2)	157 (12)
25 - 29	63 (7.9)	72 (9.05)	63 (8)	74 (9)	55 (7.9)	67 (9.5)	95 (7.9)	119 (10)
30 - 34	76 (7.6)	74 (7.4)	23 (7.6)	92 (7)	61 (7.6)	62 (7.7)	46 (7.6)	47 (8)
35 - 39	50 (7.1)	48 (6.9)	36 (7.1)	35 (7)	57 (7.1)	12 (7.4)	50 (7.1)	52 (7.4)
40 - 44	26 (6.6)	5.8(23)	13 (6.6)	12 (6)	13 (6.6)	22 (6.2)	20 (6.6)	19 (6.4)
44 - 49	18 (6)	14 (4.8)	6 (6)	5 (5)	24 (6)	5 (5.5)	30 (6)	28 (5.6)
50 - 54	37 (5.4)	30 (4.3)	16 (5.4)	13 (4)	5 (5.4)	8 (4.7)	11 (5.4)	10 (4.8)
55 - 59	23 (4.5)	17 (3.5)	18 (4.5)	14 (4)	9 (4.5)	3 (3.8)	5 (4.5)	4 (3.9)
60 - 64	22 (3.7)	13 (2.6)	4 (3.8)	3 (2.8)	4 (3.7)	3 (2.9)	4 (3.7)	3 (3)
65 - 69	3(3)	3 (2.7)	9 (3)	8 (2.6)	3 (2.9)	3 (2.5)	3 (2.9)	3 (2.5)
70 - 74	7 (2.2)	7 (2.6)	2 (2.2)	3 (2.6)	15 (2.2)	19 (2.7)	9 (2.2)	11 (2.7)
75 - 79	4 (1.5)	5 (1.6)	1 (1.5)	3 (3.5)	6 (1.5)	7 (1.8)	3 (1.5)	4 (1.9)
80 - 85	3 (0.9)	3 (0.9)	2 (0.9)	2 (0.95)	5 (0.9)	5 (1)	1 (0.9)	1 (1.1)
85 <	1 (0.4)	1 (0.2)	1 (0.4)	1 (0.3)	1 (0.4)	1 (0.3)	1(0.4)	1(0.4)

Most drowning incidents affected males (81%). A gender difference associated with the risk for drowning was 4 times higher in the male population than that in females as observed during the post-implementation period. Rivers were the most common sites for drowning, accounting for 79% of all drowning fatalities this prevalence was evidenced throughout the implementation period (Table 2).

**Table 2:** Case Related to Drowning Before and After the Program Intervention in Rural Setting of Caspian Sea Coastline (Guilan and Mazandaran Provinces)

	Baseline Study 2005, No.	Intervention Period, No.		
		2006	2007	2008
**Gender**				
Male	94	52	80	83
Female	19	16	30	7
**Location**				
River	85	55	87	70
Lake	11	11	13	11
Canal	10	0	5	0
Others	7	2	5	9
**Total**	113	68	110	90

## 5. Discussion

The principal positive finding of this study was a distinct reduction of fatal drowning during the study period. It was difficult to measure the effectiveness of the individual intervention components, separately (14). Therefore, the evaluation was performed considering the effect of the whole package. The result of baseline time of this study showed that drowning rate in intervention area was similar to that of reported from known high-risk populations in Alaska, and low- and middle-income countries in the Americas and other low- and middle-income countries in Eastern Mediterranean region (15, 16). This study identified a reduction of fatal drowning during the post-implementation period compared to pre-implementation period in the rural setting of Caspian Sea coastline. The finding from this study was consistent with the decline in the rate of drowning observed in high-income countries (17, 18). In our study, the comparison between dome-shaped pattern in the Caspian Sea beaches (19) -where the majority of drowning took place during the warmer months-and dome- shaped pattern in the rural setting -where the majority of drowning took place in rainy season when waters level in river and lake are high- showed that the drowning rate was not related to the climate. This situation was not comparable with previous studies (20-22) describing that the increase or decrease in the number of deaths was related to the seasons. It was possible that other factors might effect on the rate of drowning cases in the study areas during the study period. Consequently, multiple prevention measures were implemented, in conjunction with each other, to develop an intervention package consisting of both “passive” environmental or supervision solutions and “active” behavioral solutions. The first factor involved was the focus on encouraging communities to deliver a water-safety program, on the basis of a “community champion” model. As a result, innovative interventions need to focus on protecting and preventing children from falling in as well as on the potentials for effective post-event resuscitation actions. Our study suggests that health providers (Behvarz) and volunteers from the Red Crescent Society would benefit from CPR certification. With regard to risk factors associated with age in rural setting, this study would suggest that physical proximity was the only protective form of supervisory behavior (compared to visual or auditory supervision) for children. In exploring the nature of pre- and post-event actions, assessment of existing canal waterway fencing and lake grill legislation could be done to reduce the risk of drowning incidents, especially in young children. Like other population-based programs (23-25), this study had a number of limitations. Inadequate budgets limited the evaluation designs and activities. The most significant limitation waste lack of comparison data, constraining conclusions about association between the program and the observed changes in the impact and outcome measures. Finally, because of defects in estimation of tourist population, the rate of drowning was not calculated for this group. Selection of a before-after study protocol usually was employed because the study resources were an issue that might convey confounding factors that were difficult to control. Despite some methodological limitations, our study demonstrated that a prevention program for drowning could be effective and sustainable, when high quality local drowning data would be employed to target and model community-based injury prevention and evaluate the outcome. Our research suggested that implementing passive interventions were effective in reducing fatal drowning rate in rural areas near the Caspian Sea coastline. The results of our studies showed that drowning continues to have a significant impact on the northern Iranian community and its healthcare system. 
